# Safety and efficacy evaluation of intracerebroventricular human neural stem cell transplantation in SOD1 mice as a novel approach for ALS

**DOI:** 10.1186/s12967-025-06529-9

**Published:** 2025-05-09

**Authors:** Ivan Lombardi, Clelia Ferrero, Edvige Vulcano, Daniela Maria Rasà, Maurizio Gelati, Diego Pastor, Rose Mary Carletti, Silvia de la Morena, Daniela Celeste Profico, Sabrina Longobardi, Elisa Lazzarino, Elisa Perciballi, Jessica Diana Rosati, Salvador Martinez, Alessandro Vercelli, Angelo Luigi Vescovi, Marina Boido, Daniela Ferrari

**Affiliations:** 1 School of Medicine and Surgery, University of Milano-Bicocca, Milan, Italy; 2 Department of Biotechnology and Biosciences, University of Milano-Bicocca, Milan, Italy; 3 Neuroscience Institute Cavalieri Ottolenghi (N.I.C.O.), University of Turin, Turin, Italy; 4 Department of Neuroscience “Rita Levi Montalcini”, University of Turin, Turin, Italy; 5 University School for Advanced Studies IUSS Pavia, Pavia, Italy; 6 Production Unit of Advanced Therapies (UPTA), Fondazione IRCCS Casa Sollievo della Sofferenza, San Giovanni Rotondo, Italy; 7 Sport Research Centre, Miguel Hernández University, Avinguda de la Universitat d’Elx, Elche, Spain; 8 Cellular Reprogramming Unit, Fondazione IRCCS Casa Sollievo della Sofferenza, San Giovanni Rotondo, Italy; 9 UniCamillus - Saint Camillus International University of Health Sciences, Rome, Italy; 10 Instituto de Neurociencias de Alicante (UMH-CSIC), Universidad Miguel Hernandez, San Juan, Alicante, Spain; 11 Faculty of Medicine, Link Campus University, Rome, Italy; 12 Abu Dhabi Stem Cell Centre, Abu Dhabi, United Arab Emirates

**Keywords:** Cell therapy, Neural stem cells, Transplantation, ALS, Intracerebroventricular, SOD1 model

## Abstract

**Background:**

Neural stem cell (NSC) transplantation holds promising therapeutic potential for neurodegenerative disorders like amyotrophic lateral sclerosis (ALS). However, pre-clinical studies and early-phase clinical trials have faced challenges hindering the effective clinical translation of this approach. Crucial hurdles include the side-effects of prolonged immunosuppression, concerns regarding cell origin and transplantation dosage, identification of the most appropriate therapeutic window, and invasiveness of surgical procedures. Here, we assessed the safety and efficacy of intracerebroventricular (ICV) hNSC transplantation as a novel and possibly more effective experimental approach for ALS.

**Methods:**

We evaluated the safety of administering up to 1 × 10^6^ hNSCs in immunodeficient mice and assessed their potential efficacy in reducing ALS hallmarks employing the SOD1^G93A^ mouse model. Both transient (15 days) and prolonged immunosuppression regimens, at low (15 mg/kg) and high (30 mg/kg) doses, were tested along with two different cell dosages (3 × 10^5^ and 1 × 10^6^).

**Results:**

Our study suggests that: (i) a bilateral ICV transplantation of 1 × 10^6^ hNSCs is safe and non-tumorigenic in immunodeficient hosts; (ii) sustained and high-dose immunosuppression is essential for ensuring cell survival in immunocompetent SOD1^G93A^ mice; and (iii) hNSCs may delay motor symptom progression and reduce spinal cord microgliosis in SOD1^G93A^ mice when administered in the lateral ventricles under prolonged high-dose (30 mg/kg) immunosuppression.

**Conclusions:**

ICV transplantation of hNSCs emerges as a safe and promising strategy for ALS, demonstrating potential to delay motor decline and reduce spinal cord microgliosis. However, sustained high-dose immunosuppression is crucial for therapeutic efficacy, emphasizing the need for further optimization to overcome translational challenges and achieve durable clinical benefits.

**Supplementary Information:**

The online version contains supplementary material available at 10.1186/s12967-025-06529-9.

## Background

Amyotrophic lateral sclerosis (ALS) is a complex, multi-factorial, and progressive neurodegenerative disorder [1]. It is characterized by the progressive degeneration of both upper motor neurons (MNs), in the motor cortex, and lower motor neurons, in the brainstem and spinal cord (SC) [1]. Pathological hallmarks include MN degeneration and widespread neuroinflammation, both contributing to disease progression, culminating in extensive muscle denervation, paralysis, and death mostly from respiratory failure within 2–5 years of diagnosis [1]. While the exact etiology remains elusive, 5–10% of ALS cases exhibit a familial, autosomal dominant inheritance pattern [2] (fALS) linked to mutations in over 50 genes [3], including *TARDBP* (~ 4% of fALS), *FUS* (~ 5% of fALS), *SOD1* (~ 20% of fALS), and *C9ORF72* (~ 40% of fALS). Yet approximately 90% of ALS cases are sporadic (sALS), with unknown origin.

Several mechanisms have been proposed to contribute to ALS pathology. Among others, excitotoxicity, disruptions in cytoskeletal dynamics and axonal transport, altered RNA homeostasis, and non-neuronal cell function were demonstrated to contribute to neurodegeneration [4]. While proposed mechanisms shed light on ALS onset and progression, they also underscore the intrinsic challenges in developing effective therapies. Currently, treatment options are limited to symptomatic relief, with only few drugs offering modest survival benefits [5].

Significant efforts have been directed toward developing in vitro [6] and in vivo [7] models to uncover promising therapeutic targets for ALS. Yet, the transition to effective therapies remains challenging. Stem cell-based approaches, especially those involving neural stem cells (NSCs), have shown great promise in delaying disease progression and reducing neuroinflammation [8]. NSCs have the unique ability to integrate into the central nervous system (CNS) parenchyma, form functional neuronal connections, and provide neuroprotective, anti-inflammatory paracrine effects [8, 9]. Several pre-clinical and clinical studies have yielded encouraging results [10], highlighting the potential of these cells in ameliorating ALS hallmarks and promoting transient positive outcomes towards reduced neuroinflammation and sustained motor function. However, key hurdles, including optimal cell source and dosage, transplantation timing, injection site, and immunosuppressive regimen remain unclarified aspects [11]. Thus, non-clinical studies remain paramount to understanding ALS pathophysiology and uncovering the therapeutic potential of transplanted cells.

In a previous study [12], we demonstrated that intraspinal transplantation of clinical-grade human-NSCs (hNSCs) in a rat model of ALS (SOD1^G93A^) can target multiple disease mechanisms, resulting in significant MN preservation, reduced neuroinflammation, and extended animal survival [12]. Among others, these preclinical data have paved the way for translating this approach into clinical settings. Several Phase I studies have successfully demonstrated positive, albeit transient, effects of hNSC transplantation in ALS patients [13–15]. Nonetheless, the limited size of enrolled patient cohorts often prevents from drawing definitive conclusions.

Subsequent Phase II cell-dose-escalation studies have been conducted using the same strategy [11, 15, 16]. However, significant safety concerns and physical limitations have emerged. Specifically, the number of SC injections needed to increase the total transplanted hNSC count is constrained by the risk of backbone instability following surgery [11, 16]. To address these limitations, we opted to implement an intracerebroventricular (ICV) transplantation strategy previously adopted for secondary progressive multiple sclerosis (SPMS) patients [17]. This approach involves transplanting hNSCs into the lateral ventricles via a standardized and less invasive surgical procedure. Importantly, this strategy may facilitate the delivery of a higher number of cells and a broader distribution throughout the neuraxis. Therefore, this study aims to outline challenges and promises of optimizing a non-clinical protocol to evaluate both safety and efficacy of hNSC ICV delivery as a novel and potentially more effective treatment for ALS. Here, we investigated the effects of unilateral and bilateral ICV transplantations using hNSCs at two different dosages: 3 × 10^5^ and 1 × 10^6^ cells. Both transient (15 days) and prolonged immunosuppression regimens, administered at low and high doses, were employed.

## Methods

### Cell culture

The hNSC line used in this study was derived from a brain specimen of a single foetal donor from a spontaneous miscarriage at approximately 16 weeks post conception. The immortalized hNSC line production has been described in a previous study by our group [18]. Cell lines were isolated, expanded, and characterized according to the Neurosphere Assay as previously described [18, 19]. Briefly, after thawing, hNSCs were seeded at a density of 10^4^ cells/cm^2^ in a medium containing Epidermal Growth Factor and Fibroblast Growth Factor-2 in 25 (Corning, #CLS430639) or 75 (Corning, #CLS430641U) cm^2^ flasks (6 or 12 ml culture media, respectively), maintained in an incubator at 37 °C with > 90% humidity, 5% O_2_ and 5% CO_2_, and allowed to proliferate as free-floating clusters (neurospheres) until they reach a diameter of ~ 100 μm. At each sub-culturing passage, neurospheres were transferred to a 15 ml tube, centrifuged, the supernatant was discarded and the cell pellet mechanically dissociated through a p200 pipette in a volume of 150–200 µl to obtain a single cell solution. Cells were counted using a Burker Chamber and the viability was evaluated using the standard Trypan blue exclusion method. Finally, the single cell suspension was replated at the same initial density. This step was routinely repeated to expand the cell culture. In order to prepare cells for the transplantation procedure, 24/48 h in advance, cells were seeded at a density of 10^4^ cells/cm^2^. On the transplantation day, hNSCs were harvested by centrifugation, and viable cells were counted by Trypan blue exclusion criteria. The appropriate cell number was then re-suspended in Hanks’ Balanced Salt Solution (HBSS, Carlo Erba, #FA30WL0607500) at a density of 10^5^ cells/µl and kept in ice throughout the transplantation procedure.

### Animals

Two distinct animal models were used in this study. Adult female nude mice (Hsd: Athymic Nude-Foxn1^nu^, Envigo, #408761) were employed to assess hNSC safety and biodistribution. The maintenance of the animal colony and behavioural assessments were conducted at the Department of Biotechnology and Biosciences, University of Milano-Bicocca, Italy. For efficacy studies, adult male SOD1^G93A^ transgenic mice (B6SJL-Tg(SOD1*G93A)1Gur/J; The Jackson Laboratory, #002726) were used, except for pilot experiments (EXP 1 and 2) where male and female mice were included. The maintenance of the animal colony and behavioural tests were conducted at the Neuroscience Institute Cavalieri Ottolenghi (N.I.C.O.), Department of Neuroscience “Rita Levi Montalcini”, University of Turin, Italy. Hemizygous transgenic progeny was obtained and maintained by crossbreeding SOD1^G93A^ transgenic males with wild type females (B6SJLF/1). Female mice were previously produced by crossing a C57BL/6J (B6) female mouse and an SJL/J (SJL) male mouse. Genotyping was performed by Polymerase Chain Reaction (PCR) on DNA extracted from mouse tails at approximately 21 days of age and by incubating a 0.5 cm long specimen of tail in 100 µL of lysis buffer (10 mmol/L Tris HCl, 50 mmol/L KCl, 0.01% gelatin, 0.45% IGEPALCA-630 (Sigma-Aldrich), 0.4% Tween-20) and 25 mg of proteinase K at 55°C overnight under gentle shaking. PCR allowed to detect the presence of *hSOD1* gene. The used primers were: 5’-CAT CAG CCC TAA TCC ATC TGA-3’, Transgene Forward, and 5’-CGC GAC TAA CAA TCA AAG TGA-3’, Transgene Reverse, for the *hSOD1* gene, and 5’-CTAGGC CAC AGA ATT GAA AGA TCT-3’ and 5’-GTA GGT GGA AAT TCT AGC ATC ATC C-3’ for mouse interleukin 2 gene (*mIL-2*), as, respectively, Internal Positive Control Forward, and Internal Positive Control Reverse. All animals were maintained in a virus and antigen-free facility with controlled temperature (18–22 °C), humidity (40–60%), and 12-hour light-dark cycle. Food and water were provided *ad libitum*. All efforts were made to use the fewest number of animals and to minimize their suffering.

### Intracerebroventricular (ICV) hNSC transplantation

On the day of hNSC transplantation, mice were anesthetized with a mix of 3% isoflurane, O_2_, and N_2_O in a 1:3 ratio administrated via a gas tube. Before injection, the skin was disinfected by Betadine solution and the skull exposed by performing a midline sagittal incision with a scalpel. The correct alignment of the head (‘*flat-skull position*’) was assessed by measuring and matching the dorso-ventral coordinates of Bregma and Lambda suture under surgical microscope guidance (Zeiss). Then, and according to the different experimental purposes, mice were transplanted unilaterally (3 × 10^5^ cells/3 µl) or bilaterally (2 injections each, 5 × 10^5^ cells/5 µl) into the lateral ventricles (anteroposterior: -0.1 mm; lateral: ± 0.8 mm; dorsoventral: -2 mm from Bregma) with either hNSCs or HBSS as control through the assistance of an automatic stereotaxic apparatus (Stoelting Co.), using a manually controlled Hamilton syringe (Cemented needle, Gauge 30, length 1 cm, point style 4, 45°) at a rate of 0.5 µl/30 sec. The needle was introduced through a 2 mm diameter hole, performed in the skull by using a specific micro-drill under surgical microscope guidance to indentify the dura mater. Prior to hNSCs loading, the syringe was flushed with HBSS solution and then loaded with 3–5 µl of hNSCs suspension in HBSS (single cells resuspended to a final concentration of 1 × 10^5^ cells/µl). Post-injection, the needle was carefully removed after three minutes, and the incision was sutured with surgical clips. Mice were returned to their cages immediately after the procedure.

For safety and biodistribution analysis a total of *n* = 3 adult (5–8 weeks old) nude mice were used. For efficacy studies, at post-natal day 70 (P70; 0 days post-transplantation, dpt), mice were randomized into two experimental groups, hNSCs-treated and HBSS-treated (control group), and cyclosporin A (CsA; Sandimmune, Novartis) was administered to all animals subcutaneously on daily basis at 15 or 30 mg/kg starting from one day prior transplantation. To optimize the immunosuppression protocol, we based our initial approach on the immunosuppressive drug, dosage, and administration method previously reported in studies from our group using the same hNSC lines [12, 20] (EXP 1: 15 mg/kg CsA for 15 days). Notably, here CsA was delivered via injection rather than oral administration to prevent variability in drug intake due to possible fluctuations in water consumption. However, given the minimal hNSC survival observed under this regimen, we increased the dosage to 30 mg/kg for 15 days in EXP 2–4, approaching the maximum recommended dose for this animal model [21]. Finally, since hNSC survival was detected only during the CsA administration period, we further extended the immunosuppression regimen to 30 mg/kg until sacrifice in EXP 5.

**Fig. 1:**
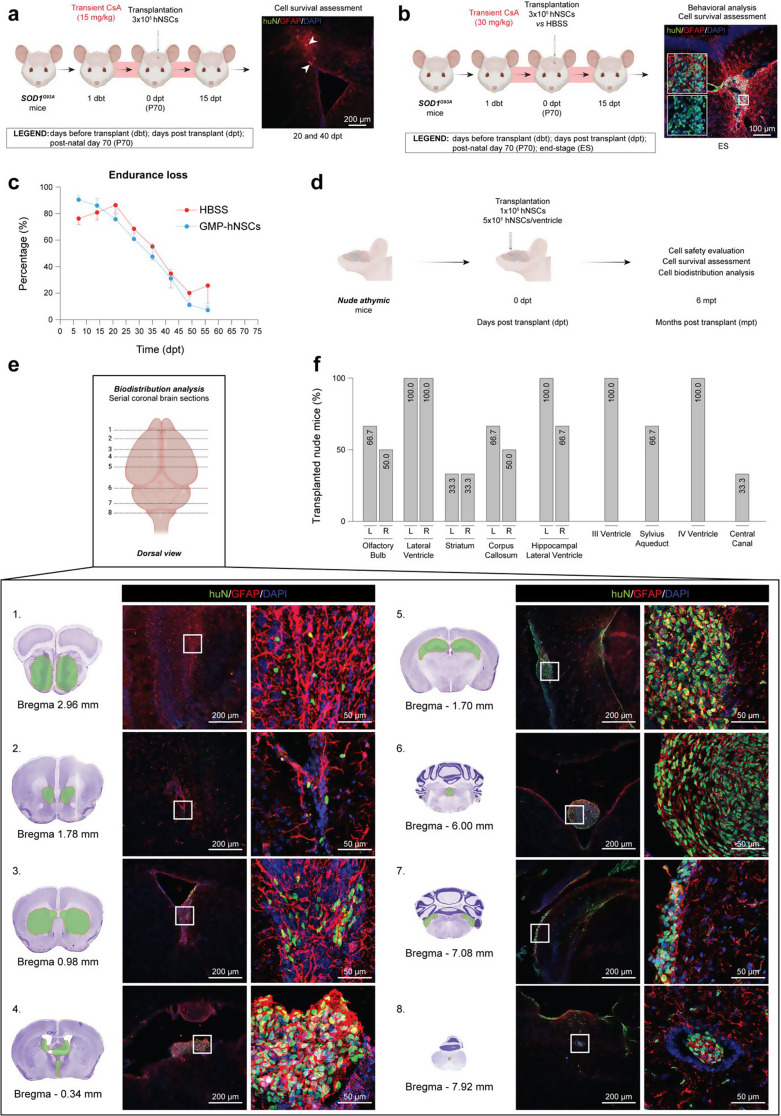
Preliminary evaluation of hNSC survival and efficacy under low- and high-dose transient immunosuppression, and long-term safety study of high-dose immortalized-hNSCs. **a**) Experimental scheme showing unilateral hNSC transplantation (3 × 10^5^) timeline into the lateral ventricle of *n* = 5 ALS mice (EXP 1) sacrificed at 20 (*n* = 2) and 40 (*n* = 3) dpt under low-dose (15 mg/kg daily) transient (15 days) immunosuppression (left) and representative confocal image of mouse brain showing only cellular debris (right)| huN, green; GFAP, red; DAPI, blue. **b**) Experimental scheme showing unilateral hNSC transplantation (3 × 10^5^) timeline into the lateral ventricle of *n* = 12 ALS mice under high-dose (30 mg/kg daily) transient (15 days) immunosuppression (left) (EXP 2) and representative confocal images of mouse brain showing huN^+^ living cells long-term (right)| huN, green; GFAP, red; DAPI, blue. **c**) Endurance loss visualization of motor performance decline considering a total of *n* = 12 mice + 3 × 10^5^ hNSCs (blue line) vs. *n* = 11 mice + HBSS (red line) (EXP 2). Two-way ANOVA, uncorrected Fisher’s LSD test, Mixed-effect model. Data are expressed as mean values ± SEM. **d**) Schematic showing the timeline of a bilateral ICV transplantation of 1 × 10^6^ immortalized-hNSCs in *n* = 3 immunodeficient mice. **e**) Upper panel: schematic representation of the different brain areas in which huN^+^ viable cells have been identified.| Lower panel: representative coronal brain sections from Paxinos et al., 2001 and confocal images of surviving huN^+^ (green) grafts 6 mpt.| huN, green; GFAP, red; DAPI, blue. **f**) Chart validating both the transplantation and cell biodistribution symmetry after bilateral injections in *n* = 3 nude mice. Data are expressed as percentage (%) over the total transplanted animals. Drawings on this figure were created with BioRender

**Fig. 2:**
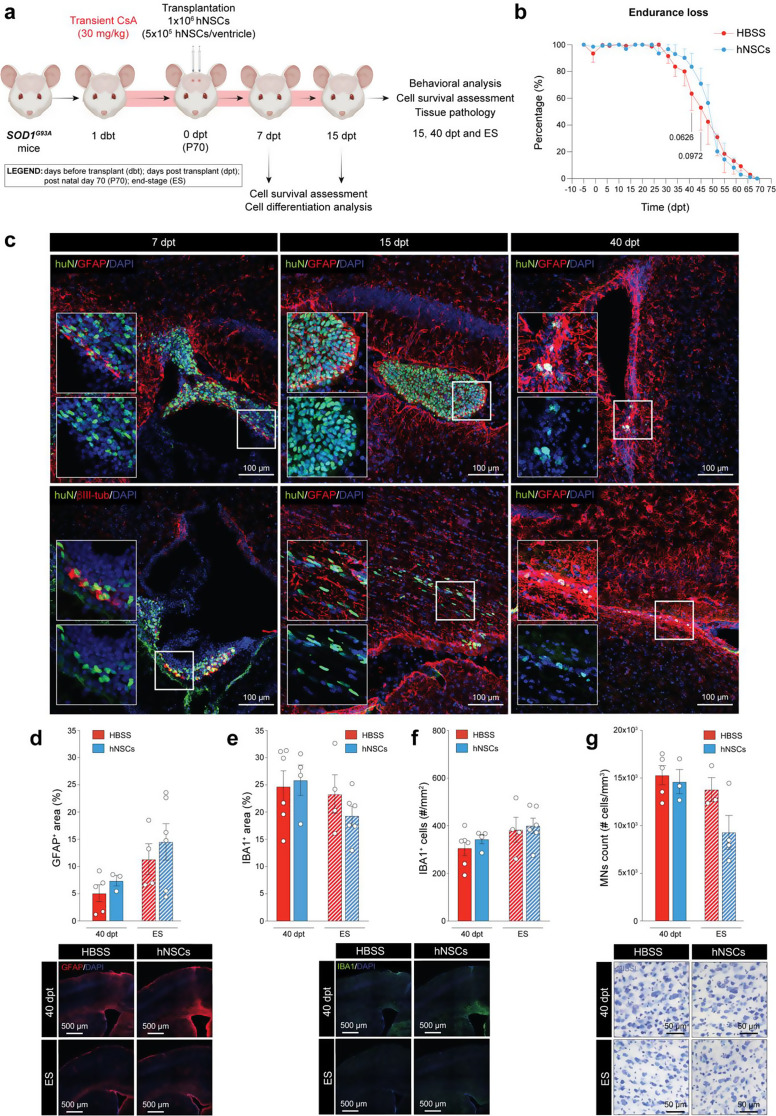
hNSC survival probability assessment and exploratory efficacy analysis of a bilateral ICV transplantation of high-dose hNSCs under high-dose transient immunosuppression. (**a**) Schematic showing the experimental setup of a bilateral high-dose (1 × 10^6^) hNSC ICV transplantation under high-dose (30 mg/kg daily) transient (15 days) immunosuppression regimen in SOD1^G93A^ mice from EXP 3 and 4 as well as *n* = 2 at both 40 dpt and ES. (**b**) Endurance loss visualization of motor performance decline considering a total of *n* = 6 SOD1^G93A^ mice + hNSCs (blue line) vs. *n* = 6 SOD1^G93A^ mice + HBSS (red line) (EXP 3); Two-way ANOVA, uncorrected Fisher’s LSD test, Mixed-effect model. (**c**) Representative confocal images showing the time-course assessment of post-transplantation hNSCs viability and differentiation profile under a high-dose transient immunosuppression regimen at 7 (*n* = 2), 15 (*n* = 2), and 40 (*n* = 6) dpt.| huN, green; GFAP, red; DAPI, blue. (**d**) Neuropathological analysis of the M1-V layer’s astrogliosis at both 40 dpt and ES on hNSC- vs. HBSS-treated mice; *n* ≥ 3/time point/group (EXP 3 and 4); parametric unpaired t-test with Welch’s correction| GFAP, red; DAPI, blue. (**e**) Neuropathological analysis of the M1-V layer’s %Area of IBA1^+^ signal at both 40 dpt and ES on hNSC- vs. HBSS-treated mice; *n* ≥ 3/time point/group (EXP 3 and 4); parametric unpaired t-test with Welch’s correction| IBA1, green; DAPI, blue. (**f**) Neuropathological analysis of the M1-V layer’s assessed counting the total number of microglial cells per mm^2^ on hNSC- vs. HBSS-treated mice at both 40 dpt and ES; *n* ≥ 3/time point/group (EXP 3 and 4); nonparametric unpaired t-test with Mann-Whitney’s correction and parametric unpaired t-test with Welch’s correction for mice at 40 dpt and ES, respectively| IBA1, green; DAPI, blue. (**g**) Upper MN stereological count in hNSC- vs. HBSS-treated mice at 40 dpt and ES; *n* ≥ 3/time point/group (EXP 3 and 4); parametric unpaired t-test with Welch’s correction. All data are expressed as mean values ± SEM. Drawings on this figure were created with BioRender

**Fig. 3:**
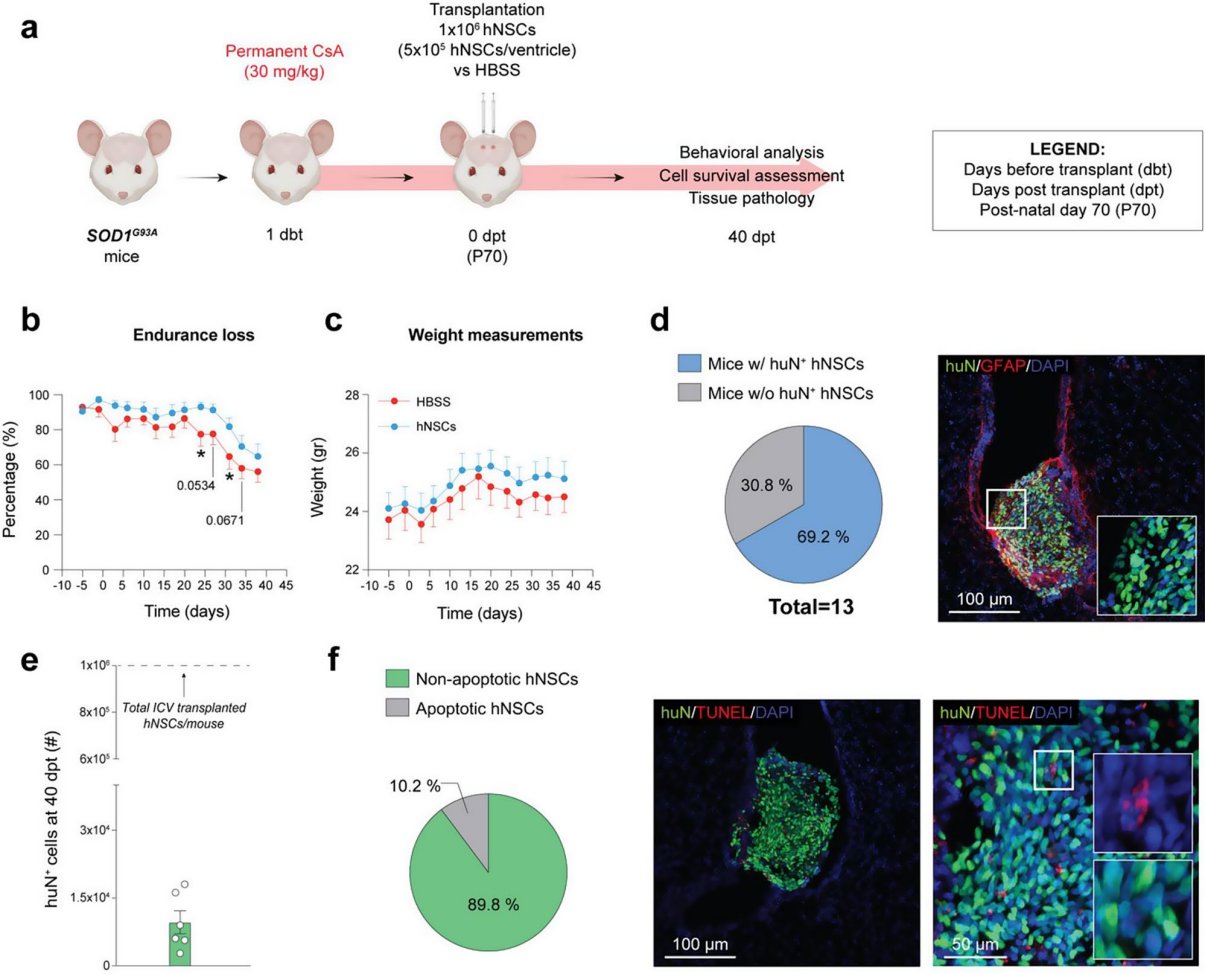
Exploratory efficacy analysis and characterization of hNSC engraftment following bilateral ICV transplantation under high-dose prolonged immunosuppression. **a**) Schematic showing the experimental setup of a high-dose hNSC bilateral ICV transplantation under high-dose prolonged immunosuppression (EXP 5) in *n* = 13 SOD1^G93A^ mice. **b** - **c**) Endurance loss visualization of motor performance decline (**b**) and weight measurements (**c**) of hNSC- (*n* = 13, blue line) vs. HBSS-treated SOD1^G93A^ mice (*n* = 11, red line); Two-way ANOVA, uncorrected Fisher’s LSD test, Mixed-effect model (p-value: *≤0.05). **d**) Pie chart showing the percentage of animals retrieved with viable hNSC grafts over the total treated animals (*n* = 13), and representative confocal images of huN^+^ engrafted hNSCs at 40 dpt.| huN, green; GFAP, red; DAPI, blue. e) Quantification of the absolute number of engrafted hNSCs in the entire brain of SOD1^G93A^ mice (*n* = 6) at 40 dpt. f) Pie chart and representative confocal images showing the relative percentages of non-apoptotic (huN^+^/TUNEL^−^) and apoptotic (huN^+^/TUNEL^+^) engrafted hNSCs over the total huN^+^ cells in *n* = 3 SOD1^G93A^ mice at 40 dpt.| huN, green; TUNEL, red; DAPI, blue. All data are expressed as mean values ± SEM. Drawings on this figure were created with BioRender

**Fig. 4:**
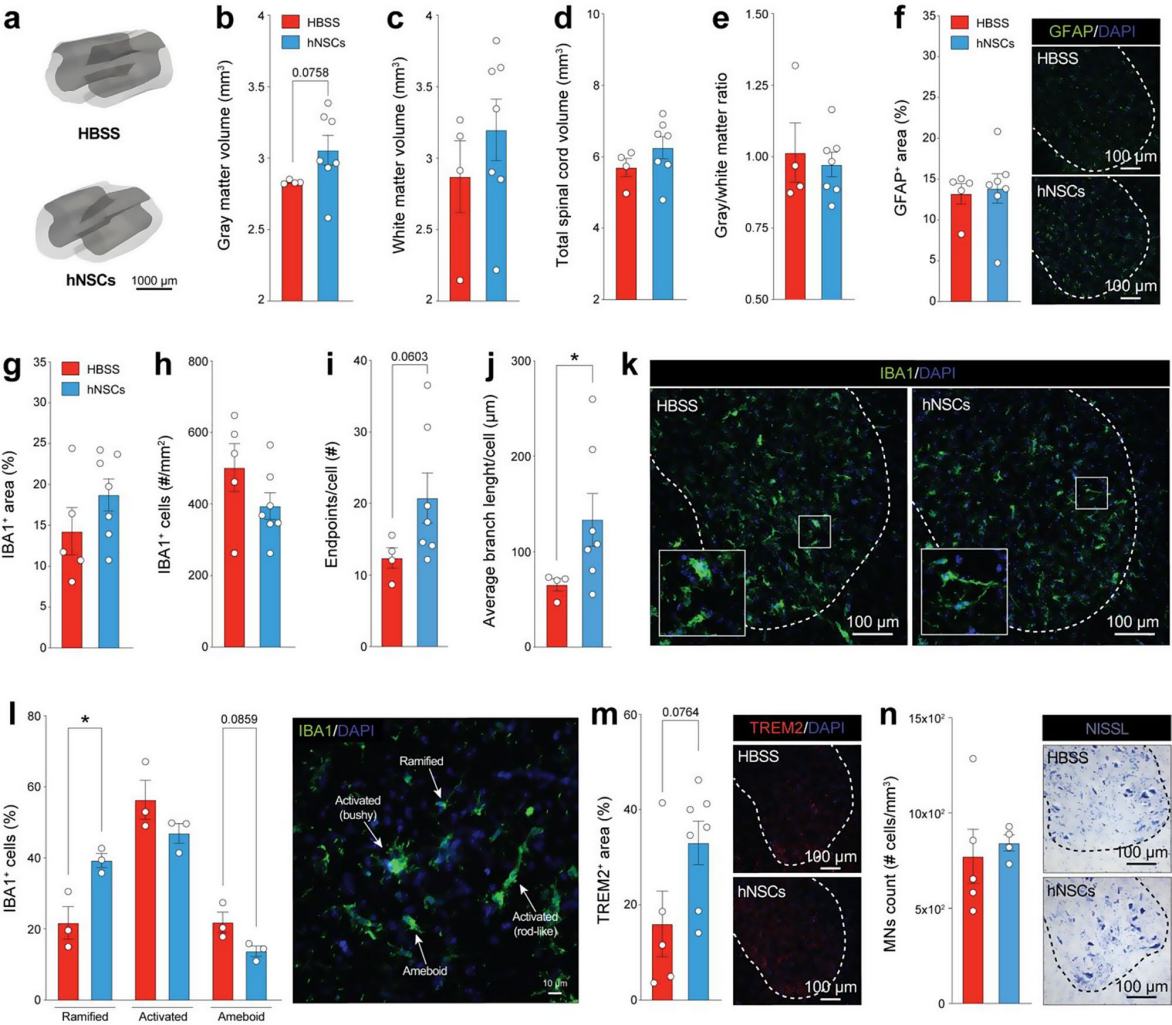
Histopathological analysis of the cervical spinal cord in SOD1^G93A^mice following hNSC ICV transplantation. **a**) Representative 3D reconstructions showing the analysed cervical SC of hNSC- vs. HBSS-treated mice (*n* ≥ 4/group; EXP 5). **b** - **e**) Analysis of (**b**) gray matter, (**c**) white matter, and (**d**) total spinal cord volume as well as **e**) gray/white matter ratio in the cervical tract of the SC of hNSC- vs. HBSS-treated mice at 40 dpt (*n* ≥ 4/group; EXP 5); parametric unpaired t-test with Welch’s correction GFAP, green; DAPI, blue. **f**) Astrogliosis evaluation of the cervical tract of the SC of hNSC- and HBSS-treated mice at 40 dpt (*n* ≥ 4/group; EXP 5); nonparametric unpaired t-test with Mann-Whitney’s correction| GFAP, green; DAPI, blue. **g**) Neuropathological analysis of the cervical tract of the SC assessed measuring microglial distribution in mice transplanted with hNSCs vs. HBSS at 40 dpt (*n* ≥ 4/group; EXP 5); parametric unpaired t-test with Welch’s correction| IBA1, green; DAPI, blue. **h**) Total amount of microglial cells (number of IBA1^+^ cells per mm^2^) in the ventral horn gray matter of the cervical SC of hNSCs- vs. HBSS-transplanted mice at 40 dpt (*n* ≥ 4/group; EXP 5); parametric unpaired t-test with Welch’s correction| IBA1, green; DAPI, blue. **i**) In vivo morphological analysis of IBA1^+^ cells at 40 dpt assessed by total number of endpoints/cell; parametric unpaired t-test with Welch’s correction (*n* ≥ 4/group; EXP 5). **j**) In vivo quantification of microglial branching at 40 dpt (*n* ≥ 4/group; EXP 5); nonparametric unpaired t-test with Mann-Whitney’s correction (p-value: *≤0.05). **k**) Representative confocal images of microglial cells within the ventral horn of the spinal cord of hNSC- vs. HBSS-treated SOD1^G93A^ mice.| IBA1, green; DAPI, blue. **l**) Phenotypic analysis and representative confocal image of microglial cells classified based on their ramified, activated (both bushy and rod-like cells), and ameboid morphology; parametric unpaired t-test with Welch’s correction (ramified and ameboid groups) and nonparametric unpaired t-test with Mann-Whitney’s correction (activated group) (*n* = 3/group; EXP 5; p-value: *≤0.05)| IBA1, green; DAPI, blue. **m**) TREM2 protein levels in the cervical tract of the SC of hNSC- and HBSS-treated animals (*n* ≥ 4/group; EXP 5); parametric unpaired t-test with Welch’s correction. **n**) MN stereological count in the cervical SC of hNSC- vs. HBSS-treated mice at 40 dpt (*n* ≥ 4/group; EXP 5); parametric unpaired t-test with Welch’s correction All data are expressed as mean values ± SEM

Animals were subdivided as follows: EXP 1 (Fig. 1a): SOD1^G93A^ mice, *n* = 5 (3 × 10^5^ cells) 15 mg/kg CsA for 15 days sacrificed at both 20 (*n* = 2) and 40 (*n* = 3) dpt; EXP 2: (Fig. 1b-c) SOD1^G93A^ mice, *n* = 12 (3 × 10^5^ cells) vs. *n* = 11 (HBSS), 30 mg/kg CsA for 15 days sacrificed at the ES; EXP 3: (Fig. 2a, b,d-g) SOD1^G93A^ mice, *n* = 6 (1 × 10^6^ cells) vs. *n* = 6 (HBSS), 30 mg/kg CsA for 15 days sacrificed at the ES; EXP 4: (Fig. 2a, c,d-g): SOD1^G93A^ mice, *n* = 6 (1 × 10^6^ cells) vs. *n* = 6 (HBSS), 30 mg/kg CsA for 15 days sacrificed 40 dpt. EXP 5: (Figures 3 and 4) SOD1^G93A^ mice, *n* = 13 (1 × 10^6^ cells) vs. *n* = 11 (HBSS), 30 mg/kg CsA for 40 days.

Finally, post-transplantation cell viability and differentiation profile were qualitatively evaluated in a total of *n* = 2 (7 dpt) and *n* = 2 (15 dpt) SOD1^G93A^ mice (1 × 10^6^ cells), 30 mg/kg CsA for 15 days (Fig. 2c).

### Behavioural and disease progression assessment

Following hNSC grafting, all transplanted animals were monitored daily and, depending on experimental needs, tested weekly or twice a week until sacrifice. A clinical checklist was adopted to detect any behavioural and neurological sign of brain damage and/or tumor formation. Key parameters included significant weight loss (> 20% relative to the start of treatment), skull deformation (hydrocephalus), exophthalmos, ataxia, and backbone curvature (kyphosis), assessed according to Guyenet et al. protocol [22]. To evaluate (i) disease progression; (ii) motor functions; and (iii) the potential efficacy of hNSC transplantation, rotarod performance was assessed, along with weight measurements, on transplanted and control SOD1^G93A^ mice starting from one week following transplantation or at P50 as described in the following paragraphs. Finally, animal survival was monitored daily until ALS mice reached their predetermined end stage. Loss of righting reflex (inability to assume a prone position within 30 s when laid on their side) or body weight loss > 20%, whichever was reached soonest, were employed to ensure ethical euthanasia and minimize suffering [23].

### Motor performance evaluation

The rotarod test was used to evaluate general motor coordination, strength, and balance [24]. We first conducted a pilot experiment (EXP 1) during which animals were placed on the rotating rod at a speed starting from a minimum of 5 r.p.m. to a maximum of 32 r.p.m. being tested once a week, starting from one-week post-surgery. In all the following behavioural experiments (EXP 2, 3, and 5), mice were first trained for 20 days prior to transplantation. Thus, animals were placed on the rotating rod at the same speed described above starting from P50 and tested twice a week. Three days post-surgery, behavioural tests continued until sacrifice day. Each session consisted of three runs, with a maximum arbitrary cut-off time of 300 s per run. The best performance time per test was used for further analysis. For statistical analysis, endurance loss was measured by dividing each mouse rotarod score at different time points by its highest recorded score.

### Weight measurements

Each mouse was weighed (in grams) once or twice a week with a standard scale until sacrifice. For subsequent analysis, the mean weight of all mice in each experimental group was calculated.

### Animal sacrifice procedure and tissue processing

To collect both brain and spinal cord (SC), animals were sacrificed by overdose of anaesthesia and perfused with 4% paraformaldehyde (PFA; Sigma #158127) in a phosphate buffer solution (PBS 1X, Sigma-Aldrich, #D8537) at different time points accordingly to experimental needs: 7, 15, 20, 40 dpt, and ES. Tissues were then removed, placed 2 h in 4% PFA at 4 °C, cryopreserved in sucrose gradients (10% for 24 h, 20% for 24 h, and 30% until the samples were fully imbibed), embedded in Optimal Cutting Temperature (OCT; Killik, Bio Optica, #05-9801) medium and frozen above liquid nitrogen vapours. To perform histological analysis, tissues were serially sectioned on a cryostat.

For the hNSC safety and biodistribution analysis in nude mice, serial coronal sections of the brain and brainstem, and transverse SC sections were cut at the thickness of 20 μm and directly placed on the surface of glass slides (epredia, #J1800AMNZ).

For SOD1^G93A^ samples, a free-floating cutting method was employed. Serial brain (coronal) and SC (transverse) sections were cut at the thickness of 30 μm and collected in a cryopreserving solution for further analysis.

### Immunofluorescence and immunohistochemistry

#### Immunofluorescence on glass slides

Coronal brain and transverse SC sections were placed on glass slides (epredia) and immunohistochemistry was performed for cell survival, differentiation, and biodistribution analysis on nude mice. Blocking Solution (PBS-1X with 10% Normal Goat Serum (NGS) and 1% TRITON X-100 was added at room temperature on each slide. Sections were then incubated in a humid chamber overnight at 4°C with the appropriate primary antibodies diluted in serum solution (NGS 10% and PBS 1X), respectively: anti-human Nuclei (huN) (1:200, Ms, IgG1, Millipore, MAB 1281), anti-Glial Fibrillary Acidic Protein (GFAP) (1:500, Rb, IgG, Dako Z0334), and anti-bIII Tubulin (1:400, Rb, IgG, BioLegend, 802001). The day after, tissues were washed with PBS 1X and then incubated for 90 minutes at room temperature with the appropriate secondary antibodies (Abs II) in serum solution, as described before. The following Abs II were used: Alexa Fluor 488 (1:1,000, α-Ms, Jackson, #115-545-205) and Alexa Fluor 594 (1:1,000, α-Rb, Invitrogen, #A11012). Cell nuclei were labelled with 4’,6-Diamidin-2-phenylindol (DAPI, 1:1,000, Roche Diagnostics GmbH, Mannheim, Germany, #10236276001) in PBS 1X for 20 min at room temperature and the slides mounted using coverslips with aqueous mounting (Fluor Save Reagent, Calbiochem, #CALB345789-20).

#### Immunofluorescence on free floating sections

Neuroinflammation analysis on SOD1^G93A^ mice was performed via immunofluorescence following the free-floating protocol. Samples were rinsed 3 times with PBS 1X on tilting plate. A permeabilization step followed with the addition of 0.3% TRITON X-100 and PBS 1X for 30 min at room temperature on tilting plate. Blocking solution (10% NGS or NDS, 0.3% TRITON X-100, PBS 1X) was added for 30 minutes at room temperature on tilting plate. Then, sections were incubated overnight at 4°C with anti-GFAP (1:500, Rb, IgG1, Dako, Z0334), anti-IBA1 (1:500, Rb, IgG, Wako, 019-19741), anti-TREM2 (1:100, Rat, IgG2b, Biotechne R&B System, MAB 17291), anti-CD86 (1:100, Rat, IgG2a, Invitrogen, 14-086282), and anti-CD206 (1:200, Goat, IgG, R&D Systems, AF2535) antibodies. The next day, after washing with PBS 1X, the following Abs II were used: Alexa Fluor 594 (1:500, α-Rat, Invitrogen, A11007 or 1:200, α-Rat, Jackson, 712-165-150), Alexa Fluor 488 (1:1,000, α-Rb, Jackson, 111-545-144), and Alexa Fluor 488 (1:200, α-Rb, Jackson, 711-545-152). Cell nuclei were labelled with 4’,6-Deamidine-2’-phenylindole dihydrochloride (DAPI, 1:1,000; Roche, #10236276001) for 20 min at room temperature. After washing with PBS 1X, samples were mounted on glass slides (epredia), left to dry and then gently washed with deionized water (dH2O). Slides were cover-slipped with aqueous mounting as before.

### Nissl staining

Brain and SC sections were Nissl-stained to perform motor neuron (MN) stereological count. Respectively, serial coronal brain or transverse SC sections were hydrated in distilled water for 1 min before staining in 0.1% Cresyl Violet acetate for 10 min, then dehydrated in an ascending series of ethanol (EtOH 95% and EtOH 100%) for 30 s, cleared in xylene for 30 s for 2 times, and cover-slipped with Eukitt (Bioptica, Milan, Italy, #W01030706). Sections were mounted on 4% gelatine-coated Superfrost slides (epredia) and air-dried overnight at room temperature.

### hNSC safety, survival, differentiation, and biodistribution analysis

The safety, differentiation, and biodistribution of hNSCs transplanted into the lateral ventricles of *n* = 3 nude mice were evaluated qualitatively by serial Z-stack confocal images of different part of the brain, brainstem, and SC.

Quantitative assessment of hNSC survival in SOD1^G93A^ mice with surviving grafts was performed on *n* = 6 animals from EXP 5 using anti-huN antibodies. A total of 32 coronal brain sections per mouse were evaluated, representing 4 out of 24 series that encompassed the entire brain. A manual count of huN^+^ cells was conducted on confocal images acquired at 20x magnification. The total number of huN^+^ cells per series was then multiplied by 6 to estimate the total number of engrafted, viable cells per animal.

Apoptotic hNSCs were quantitatively assessed in *n* = 3 animals from EXP 5 (2 series/animal for a total of 16 brain coronal sections/animal) using an in situ cell death detection kit (TUNEL assay, Roche, #11684795910). Briefly, after immunostaining with anti-huN antibodies, brain sections were incubated with the TUNEL reaction solution (enzyme solution: label solution = 1:10) for 1 h at 37 °C, followed by nuclear counterstaining with DAPI. Apoptotic cells (huN^+^/TUNEL^+^) and total huN^+^ cells were manually counted on confocal images acquired at 20x magnification. Apoptosis was expressed as a percentage using the formula: (huN^+^/TUNEL^+^ cells / total huN^+^ cells) x 100.

Nikon A1R confocal microscope was used for image acquisition.

### Motor cortex neuroinflammation analysis

All the images of serial coronal sections throughout the rostro-caudal extent of the M1 motor cortex-V layer were obtained by performing large-image acquisitions with Nikon A1R confocal microscope using the 10x objective and processed with Software NIS Elements A1 Version 5.30.06. The lambda (λ) parameter (optical configuration) was set on three channels as follows: DAPI (HV = 90, Offset = -80, Laser = 4.0), FITC (HV = 60, Offset = -80, Laser = 3.5), and TRITC (HV = 40, Offset = -70, Laser = 2.0). In order to obtain an image containing all the motor cortex (M1), the large image parameter was set to be 2 × 2 or 3 × 3, depending on ROI (Region Of Interest) dimension. Z-stack of 28 μm thickness with 10 steps sampling were acquired from the top to the bottom of each section, with an acquisition interval of 3.11 μm each. Consecutive Z-stack images were converted to a maximum intensity projection (MIP) picture and the final Z-stack confocal image was obtained for further examinations.

Quantitative neuroinflammation analysis was conducted on serial coronal brain sections of both HBSS- and hNSC-treated SOD1^G93A^ mice from EXP 3 and EXP 4 at different time points: 40 dpt and ES. Z-stack confocal images of coronal sections in TIFF format were analysed with Fiji ImageJ (ImageJ2, Version 2.9.0/1.53t) and Photoshop (23.5.0 Release). Firstly, the M1-V layer’s ROI was manually drawn on confocal images based on both DAPI staining and the additional guide of *The Mouse Brain Atlas in Stereotaxic Coordinates* [25].

For each channel, the % of Area (%Area) of the GFAP^+^ and IBA1^+^ signal was quantified on thresholded and non-thresholded images to discriminate the real active pixels from the background. For thresholded images, the %Area is the percentage of pixels in the ROI highlighted by using the “*Mean*” Threshold parameter. For non-thresholded images, the %Area is the percentage of non-zero pixels. For statistical analysis, a minimum of 3 and a maximum of 14 serial coronal brain sections/animal were analysed and the plot value/animal was calculated as follows: (sum of all the areas of the total thresholded images/ sum of all the areas of the total non-thresholded images)*100.

Additionally, the number of microglial (DAPI^+^/IBA1^+^) cells per mm^2^ was quantified and included in our analysis. A manual, non-stereological count was performed on the previously described ROIs by Count tool on Photoshop or ImageJ. To normalize the obtained data, the final plot value/animal was obtained as follows: (sum of the counted DAPI^+^/IBA1^+^ cells in each ROI / ROIs area in mm^2^).

### SC neuroinflammation analysis

Confocal images of transverse sections of the cervical (C1-C8) tract of the SC were acquired at 20x magnification using a Nikon A1R confocal microscope. Both HBSS- and hNSC-treated SOD1^G93A^ mice from EXP 5 at 40 dpt were analysed. Identical camera and fluorescent light source settings were used across all sections and for each paired antibodies. The lambda (λ) parameter (optical configuration) was set on three channels as follows: DAPI (HV = 70, Offset = -50, Laser = 5.0), FITC (HV = 50, Offset = -80, Laser = 3.5), and TRITC (HV = 32, Offset = -95, Laser = 4.0) for IBA1/TREM2 antibodies; and DAPI (HV = 70, Offset = -50, Laser = 5.0) and FITC (HV = 30, Offset = -90, Laser = 3) for GFAP antibodies. Z-stack of 15 μm thickness with 8 steps sampling were acquired from the top to the bottom of each section, with an acquisition interval of 2.14 μm each. Consecutive Z-stack images were converted to a maximum intensity projection (MIP) picture and the final Z-stack confocal image was obtained for further examinations. Four sections per cervical segment of each animal were selected for quantification analysis using Image J software.

The %Area of the GFAP^+^, IBA1^+^, and TREM2^+^ signal was measured within a constant ROI (size: 487.01 × 408.13 μm) centred in the grey matter of the ventral horn. The quantification was performed on thresholded and non-thresholded images, as previously described. The plot value/animal was calculated as follows: (sum of all the areas of the total thresholded images/ sum of all the areas of the total non-thresholded images)*100.

Moreover, microglial density (DAPI^+^/IBA1^+^ cells per mm^2^) was quantified following the same approach previously described for motor cortex neuroinflammation analysis. Additionally, microglia morphology was assessed by measuring the number of endpoints/cell and average branch length/cell in µm employing a validated Fiji ImageJ protocol for 20x immunofluorescence images [26]. First, brightness and contrast of each image was adjusted in order to clearly visualize microglia processes and maximum up to the edges of the histogram. Then, an unsharp mask filter (pixel radius = 3 and mask weight = 0.6) was applied to further improve contrast followed by a despeckle step and binary conversion using the threshold function. Noise reduction and closure of process gaps were performed, and outliers were removed using the “Remove Outliers” function (pixel radius = 0.5 and threshold = 50). A skeletonized image was then generated and analysed using the “AnalyzeSkeleton (2D/3D)” plugin in Fiji ImageJ v.2.14.0. A cut-off value of 2.583 for undesired fragments was determined by averaging the length of small artifacts identified using the line tool, ensuring consistency across all datasets. The resulting Excel file was used for data extraction, with values expressed as the average number of endpoints per cell and average process length (µm/cell) from *n* ≥ 4 ROIs (one per SC section) per mouse, across *n* ≥ 4 independent biological replicates (± SEM).

Finally, microglial activation states were assessed by three independent operators based on morphological classification (ramified, activated - both bushy and rod-like -, and ameboid, see examples in Fig. 4l) in *n* = 3 mice/group, analyzing *n* = 4 ROIs/mouse (one per SC section).

To further analyze the microglial phenotype (M1 vs. M2), the positivity to CD86, CD206 or CD86/CD206 markers was quantified by counting the percentage of labelled cells over the IBA1^+^ cell population in the ventral horns of cervical spinal cord. For each animal (HBSS, *n* = 3; hNSCs, *n* = 3), 2 images per section were acquired and a total of 60 IBA1^+^ cells per animal were counted. All fluorescence images were acquired using an Apotome 3 f/Axio Imager Z2 (Zeiss) equipped with Axiocam 705 mono R2 camera at 20x magnification: each acquisition consisted of 12 z-stacks with a step size of 1 μm. Additionally, the mean grey value (MGV) of IBA^+^, CD86^+^, and CD206^+^ cells was quantified by using ImageJ software (HBSS, *n* = 3; hNSCs, *n* = 3; 6 images/animal).

### Motor neuron stereological counting

Upper and lower motor neuron (MN) quantification was performed on both hNSC- and HBSS-treated SOD1^G93A^ mice at different time points, 40 dpt and ES. The motor cortex (M1’s layer V) and the ventral horns of cervical (C3-C5) spinal segments have been evaluated. One series of Nissl-stained serial brain and SC Sect. (30 μm-thick; one every 180 μm) from each group was analysed. Per each animal, 9 and 10 sections were counted for the motor cortex and SC, respectively. The estimation of MN density was obtained by using a stereological technique, the Optical Fractionator, with a computer-assisted microscope and the Stereo-Investigator software (MicroBrightField, Williston, VT, USA). Cells were counted on the computer screen with the use of an Optronics MicroFire digital camera mounted on a Nikon Eclipse E600 microscope. 3 μm guard zones, 12 μm optical dissector height, 100 × 100 μm counting frame size and 150 × 150 μm scan grid size were set up to perform the counts. MNs’ nucleoli were counted at 40x magnification. Only neurons with a diameter ≥ 10 μm (classified as large pyramidal cells in the cerebral motor cortex) and ≥ 16 μm (considered alpha MNs in the SC) and located in the appropriate position were counted. The total volume of the reconstructed segment (expressed in mm³) and the MN density (expressed as number of MNs/mm^3^) were calculated.

### SC volume analysis

The volume of both grey and white matter were assessed using Neurolucida software (Version 9) on 4 serial cervical SC transverse sections per animal (one every 720 μm) sampled within the cervical tract considering the cutting thickness of 30 μm. The total and grey matter volume/section per animal were calculated in mm^3^. Then, the white matter volume was estimated by subtracting the grey matter volume from the total volume for each section.

### Statistical analysis

Statistical analysis was conducted using GraphPad Prism Software (Version 9.5.0, GraphPad Software, La Jolla, CA, USA). Normal distribution of individual values was verified prior to each test, and outliers were excluded following an outliers identifier test (ROUT method, Q = 5%). For comparisons between 2 groups and > 1 condition, a Two-Way ANOVA (mixed-effect model) followed by Fisher’s LSD test was performed. For comparisons between 2 groups under 1 condition, a parametric unpaired t-test with Welch’s correction or a nonparametric unpaired t-test with Mann-Whitney’s correction was used. All data are presented as mean ± SEM (Standard Error of the Mean), and significance was determined at *p* ≤ 0.05 (*). To determine the appropriate sample size for each experimental group, we conducted an a-priori power analysis using G*Power software. This analysis was based on the expected variability observed in our previous studies employing the same mouse strain and experimental techniques [27, 28].

## Results

### Unilateral ICV injection of low-dose hNSCs under high-dose transient immunosuppression shows suboptimal cell survival in SOD1^G93A^ mice

Previous data by our group demonstrated the safety and differentiation profile of extensively characterized [29] GMP-hNSCs upon unilateral ICV transplantation in immune-deficient adult mice [17].

Starting from these results, we investigated the survival and integration of hNSC in immunocompetent SOD1^G93A^ mice under a mild-dose (15 mg/kg daily) transient (15 days) immunosuppression regimen with CsA. We transplanted a total of 3 × 10^5^ cells unilaterally at approximately 70 days of age (P70) (EXP 1), a stage when histological markers of the disease are already present, though motor symptoms are still negligible [30–32]. When sacrificed at 20 and 40 dpt we did not observe surviving cells via immunostaining for the human-specific antibody (huN). This indicates that a mild immunosuppression was insufficient for hNSC survival in mice (Fig. 1a). To improve hNSC engraftment, we employed the highest immunosuppression dosage recommended for mice (30 mg/kg daily) [21] for 15 days upon injection of the same hNSC dose (Fig. 1b, **left**). Motor functions and weight were monitored until end stage (ES). In this second experiment (EXP2), huN^+^ hNSCs were detected in 3 out of 12 ES mice, mostly near the ventricles (Fig. 1b, **right**). However, we did not observe any behavioural improvement in motor performances (Fig. 1c), weight decline or survival rate (data not shown) in transplanted SOD1^G93A^ mice vs. controls (SOD1^G93A^ mice + HBSS).

Altogether, these results suggest that, although increasing immunosuppression enhanced hNSC survival, the cell dosage was still suboptimal to contrast disease progression.

### Bilateral ICV transplantation of high-dose immortalized hNSCs is feasible and safe

We then investigated whether increasing the dosage of transplanted hNSCs could ameliorate histological and behavioural hallmarks in ALS mice. Here, we exploited the greater in vitro amplification potential, as well as promising therapeutic abilities [20, 33], of an immortalized hNSC line previously characterized in our lab [18]. We first assessed the safety of transplanting a high dose of hNSCs through a long-term study in nude athymic mice (Fig. 1d-e). A total of 1 × 10^6^ immortalized hNSCs were injected into the lateral ventricles of adult (5–8 weeks) nude mice via bilateral ICV injection (5 × 10^5^ cells/ventricle). Animals were euthanized at 6 mpt (Fig. 1d), and gross pathological analysis showed no signs of inflammatory or neoplastic processes such as alterations of the cerebral parenchyma and ventricles asymmetry. Moreover, serial brain, brainstem, and SC sections, allowed us to identify a robust migratory capacity of transplanted hNSCs, reaching the 3rd and 4th ventricles up to the central canal of the cervical SC. Of note, hNSCs were detected adhering to the ventricular wall and migrating into the parenchyma, reaching multiple brain structures such as olfactory bulbs, striatum, lateral septum, corpus callosum, and CA1 region of the hippocampus. The most rostral and caudal CNS regions in which we identified transplanted hNSCs were Bregma 2.96 mm and Bregma − 7.92 mm, respectively (based on *The Mouse Brain Atlas’* [25] annotations, Fig. 1e). Finally, we assessed the symmetry of our bilateral transplantation strategy and found no differences in hNSC biodistribution across anatomically matched regions of the left (L) and right (R) hemispheres (Fig. 1f), confirming a uniform cell delivery and distribution within the CNS.

Overall, our results demonstrate that the transplantation of a high dose (1 × 10^6^) immortalized hNSCs via bilateral ICV injection in immunodeficient hosts is feasible and safe.

### Bilateral ICV transplantation of high-dose hNSCs under high-dose transient immunosuppression shows trend in delayed motor decline

To investigate the potential benefits of high-dose hNSC transplantation, we next transplanted SOD1^G93A^ mice bilaterally with 1 × 10^6^ hNSCs at P70 under a high-dose (30 mg/kg daily) transient (15 days) immunosuppression regimen (Fig. 2a). We first assessed motor functions and animal survival until ES and observed a slight delay in motor decline in the hNSC-treated group vs. controls, particularly between 41 (84 ± 10% vs. 64 ± 13%, respectively; *p* = 0.0626) and 45 dpt (71 ± 12% vs. 53 ± 17%, respectively; *p* = 0.0972) (Fig. 2b; EXP 3), while no differences were observed in animal survival (data not shown). We then performed a qualitative time-course analysis of post-transplantation cell viability and differentiation profile (Fig. 2c). At 7 dpt, we observed both huN^+^/bIII-tub^+^ and huN^+^/GFAP^+^ hNSCs, indicating their in vivo differentiation into neuronal and astrocytic lineages. Moreover, viable huN^+^ hNSCs persisted up to 15 dpt and migrated into the brain parenchyma showing a “stretched” morphology (Fig. 2c). However, at later time points (40 dpt and ES), we observed viable hNSCs inside the third ventricle of only one ES mouse (data not shown). When huN^+^ cells were not detected, cellular debris were identified near the injection site and within the cerebral parenchyma (Fig. 2c), indicating suboptimal long-term survival of hNSCs under transient high-dose immunosuppression.

Despite the low long-term survival rate of hNSCs, we investigated whether their persistence into the host CNS was sufficient to induce motor cortex histopathological changes. We first assessed both immunofluorescence GFAP^+^ and IBA1^+^ signals, indicative of astro- and micro-gliosis, respectively, in the V-layer of the murine primary motor cortex (M1) and found no major differences both at 40 dpt and ES (Fig. 2d-f). These findings suggest that the level of hNSC persistence achieved with transient high-dose immunosuppression was insufficient to induce substantial histopathological changes in the motor cortex. Moreover, at 40 dpt and in both groups (hNSCs vs. HBSS; EXP 4), we observed a marked increase in the %GFAP^+^ area in some brain sections, predominantly near the injection site, but occasionally extending rostrally into the murine cortex (Fig. 2d, **lower 40 dpt panels**). This increase, likely resulted from the surgery-induced focal astrogliosis, potentially confounding and limiting any cortical neuropathological analysis at intermediate time points. By ES, despite the overall GFAP^+^ signal was higher (Fig. 2d), this effect was reduced, even becoming undetectable in some mice, suggesting it was transient and spatially confined (Fig. 2d, **lower ES panels**). Next, we evaluated the MN degeneration rate in the same anatomical region via stereological count and obtained comparable number of MNs between hNSC-treated and control groups at both 40 dpt and ES (Fig. 2g).

Overall, these results indicate that high-dose (30 mg/kg) transient (15 days) immunosuppression did not ensure optimal cell survival following high-dose (1 × 10^6^) hNSC transplantation. However, a promising trend of transient behavioural improvement was observed around 40 dpt, despite persistent astro- and microgliosis and ongoing upper MN degeneration, suggesting a potential hNSC-mediated effect in delaying motor impairments. Nonetheless, further optimization of cell survival is necessary to elicit concomitant histopathological changes and achieve more sustained therapeutic outcomes.

### Bilateral ICV transplantation of high-dose hNSCs under high-dose prolonged immunosuppression delays motor symptoms progression

We next tested whether an increased dose (30 mg/kg daily) and extended duration (until sacrifice) of immunosuppression would enhance behavioural and histopathological outcomes in SOD1^G93A^ mice following ICV transplantation of 1 × 10^6^ hNSCs (EXP 5; Fig. 3a). Focusing on animals sacrificed at 40 dpt, an intermediate time point where prior studies [12] suggest the greatest pathological amelioration, and consistent with results previously shown in Fig. 2b, we observed significantly sustained motor function in the hNSC-treated group vs. controls starting from 24 dpt (93 ± 3% vs. 78 ± 7%, respectively) and lasting until 31 dpt (82 ± 5% vs. 65 ± 7%, respectively) **(**Fig. 3b**)** without affecting weights in both groups (Fig. 3c). Hence, we investigated cell engraftment and viability. We detected huN^+^ hNSCs in 9/13 (~ 70%) transplanted animals, extending from the lateral ventricles to the aqueduct of Sylvius and 4th ventricle (Fig. 3d). When investigating the total number of surviving hNSCs, we found 9653 ± 2519 huN^+^ cells, accounting for 0.96 ± 0.25% of the total transplanted cells (1 × 10^6^) (Fig. 3e). Additionally, the TUNEL assay showed that 10.2% of the huN^+^ cells was apoptotic (Fig. 3f), indicating that most of the successfully engrafted cells remained viable at 40 dpt within the unfavorable, diseased environment.

These findings suggest that extended immunosuppression enables sufficient hNSC survival, contributing to significant motor improvements.

### Bilateral ICV transplantation of high-dose hNSCs under high-dose prolonged immunosuppression favours microglial ramification in the cervical spinal cord

We then assessed whether hNSCs transplanted under prolonged immunosuppression induced histopathological changes. Here, we focused our analysis on the cervical and lumbar SC tracts, both described as particularly vulnerable regions to neurodegeneration in ALS pathology [34, 35]. Importantly, this approach also avoids the confounding surgery effects seen in our earlier motor cortex analysis. We first measured gray (3.06 ± 0.10 mm^3^, hNSCs vs. 2.83 ± 0.006 mm^3^, HBSS; *p* = 0.0758) and white matter (3.20 ± 0.21 mm^3^, hNSCs vs. 2.87 ± 0.25 mm^3^, HBSS) volumes of SOD1^G93A^ mice and found a trend toward reduction in cervical SC degeneration in hNSC-transplanted mice vs. controls (Fig. 4a-c). While the total cervical SC volume in the hNSC group showed a non-significant trend indicative of lower parenchymal degeneration vs. controls (6.25 ± 0.31 vs. 5.70 ± 0.25 mm^3^, respectively), the gray-to-white matter ratio remained consistent across groups, potentially indicating comparable disease effect in both areas (Fig. 4d-e). Overall, these findings might suggest a potential for hNSC-induced neuroprotection in mitigating cervical SC degeneration as indicated by trends in gray and white matter volume preservation.

To investigate a potential neuroprotective mechanism exerted by transplanted hNSCs, we assessed astro- and micro-gliosis levels in the ventral horn gray matter of the same SC tract. By looking at the GFAP^+^ astrocytes, we observed both groups to have similar astrocyte coverage suggesting that transplanting high-dose (1 × 10^6^) hNSCs under a prolonged high-dose immunosuppression did not impact astrogliosis (Fig. 4f). To investigate whether hNSC transplantation affects microgliosis, we assessed IBA1^+^ cells distribution (Fig. 4g), number (Fig. 4h), and morphology (Fig. 4i-k) in the hNSC-treated vs. control group. Interestingly, we observed a trend toward increasing IBA1^+^ %Area (18.73 ± 1.97% vs. 14.30 ± 2.87%, respectively; Fig. 4g) alongside a decrease trend in the total number of IBA1^+^ cells (394.00 ± 37.24 cells/mm^2^ vs. 501.60 ± 67.16 cells/mm^2^, respectively; Fig. 4h). This suggests that while microglial cell number is reduced, they occupy a larger area within the ventral horn gray matter, potentially reflecting reduced microgliosis and a shift toward a more ramified, anti-inflammatory, phenotype. Consistently, morphological analysis of IBA1^+^ cells revealed a greater number of endpoints per cell (20.70 ± 3.50 vs. 12.40 ± 1.44; *p* = 0.0603; Fig. 4i) and significantly longer branch lengths (133.60 ± 27.62 vs. 65.50 ± 6.26 μm; Fig. 4j) in the hNSC vs. HBSS group, respectively. This is indicative of less activated microglial morphology suggesting reduced cervical SC inflammatory environment in hNSC-transplanted ALS mice. To further explore this hypothesis, we then performed a quantitative analysis of the three main microglial states (ramified, activated, and ameboid), within the same anatomical region (Fig. 4l), as previously described in the literature [36–38]. Notably, the percentage of ramified IBA1^+^ cells was significantly higher in the hNSC group compared to controls (39.28 ± 2.01% vs. 21.74 ± 4.60%, respectively). Moreover, while the proportion of activated microglia remained unchanged between groups (56.37 ± 5.47%, hNSCs vs. 46.94 ± 2.77, HBSS), we observed an interesting yet non-significant trend towards a reduced percentage of ameboid microglia in the cervical spinal cord of SOD1^G93A^ mice + hNSCs vs. controls (21.88 ± 2.8 vs. 13.76 ± 1.46, respectively; *p* = 0.0859) (Fig. 4l), further suggesting an overall dampening of neuroinflammation. To corroborate these findings, we next assessed TREM2^+^ microglia levels within the same anatomical region (Fig. 4m). Here, we observed a trend toward increased %TREM2^+^ area compared to the HBSS control group (33.03 ± 4.5% vs. 16.03 ± 6.8%, respectively; *p* = 0.0764; Fig. 4m). This may support TREM2 crucial role in promoting a neuroprotective microglia phenotype. Next, we analyzed the expression of microglial markers CD86 and CD206 [39, 40] to identify M1 (CD86^+^IBA1^+^), M2 (C206^+^IBA1^+^) and double-labeled (CD86^+^CD206^+^IBA1^+^) phenotypes (Suppl. Figure 1a). The analysis showed no significant differences in marker fluorescence intensity (Suppl. Figure 1b) or the relative proportions of microglial cells (Suppl. Figure 1c). Finally, we examined whether the observed microglial changes were coupled with a higher MN preservation in the cervical SC (Fig. 4n). Alpha MN count did not reveal significant differences between the hNSC vs. control group. However, a non-significant trend favouring the hNSC group was observed (843.10 ± 43.03 vs. 773.40 ± 141.80 cells/mm^3^, respectively). Further experiments will be necessary to confirm the potential of hNSC-mediated neuroprotective effects on MNs. Of note, no differences were observed in the lumbar SC (data not shown), confining positive results to anatomical regions directly reached by transplanted cells.

Collectively, these findings indicate that high-dose (1 × 10^6^) hNSC transplantation in SOD1^G93A^ mice under sustained high-dose (30 mg/kg) immunosuppression mitigates endurance loss. Furthermore, despite being injected distally in the lateral ventricles, hNSCs show potential to reduce microglia activation in the cervical SC, highlighting their widespread anti-inflammatory effects.

## Discussion

Currently, no therapy can reverse or halt ALS progression. Given the severity of the prognosis, there is an urgent need to translate promising approaches into effective clinical treatments. Here, we aimed to optimize key experimental parameters to establish an alternative, non-clinical ICV transplantation protocol of hNSCs for ALS. Our findings demonstrate that under a prolonged, high-dose immunosuppression hNSCs can survive, spread along the neuraxis, and occasionally migrate into the cerebral parenchyma of SOD1^G93A^ mice. Furthermore, transplanted hNSCs delay motor symptoms degeneration and reduce cervical spinal cord microgliosis in this ALS mouse model. To our knowledge, this is the first study to show that foetal-derived hNSCs transplanted into the lateral ventricles may induce both behavioural and histological changes in ALS pathology. Moreover, our work reveals the low immune tolerance of mice to transplanted hNSCs, thus suggesting that alternative models, such as rats, may provide a more suitable environment for long-term integration studies of human cells [12].

A critical step in developing an effective stem cell therapy is identifying the optimal delivery route that can be both safe and efficient in directing transplanted cells to the target region(s). This challenge is further compounded in multifocal neurodegenerative diseases like ALS, where intra-parenchymal transplantation would necessitate multiple injections across affected regions. Despite these limitations, early preclinical studies have explored direct intraspinal hNSC delivery under stereotactic guidance for precise cell administration into the anterior horns of the spinal cord. Notably, this approach has demonstrated beneficial effects, including partial protection of spinal MNs, delayed motor function degeneration, and extended survival [12, 41–45]. Although clinical studies have partially confirmed these results, the benefits proved to be transient, likely due in part to the aggressive nature of ALS and the underestimation of optimal cell dosage [13, 14]. In this context, increasing the number of transplanted cells via multiple intraspinal injections in SOD1 mice has been reported to cumulatively enhance therapeutic effects [43], thus reinforcing the rationale that suboptimal dosing may limit hNSC efficacy. However, translating this strategy to a broader clinical practice is hindered by the risks associated with the extensive laminectomy and invasiveness required for multiple SC injections in humans.

Alternatively, previous non-clinical studies have explored the safety and feasibility of systemic NSC delivery via intravenous (IV) injection or direct administration into the CSF through ICV (mainly intra-cisterna magna) or intrathecal (IT) routes in preclinical models of inflammatory CNS disorders [46]. Among these, IV injection is considered the safest, as it meets WHO standards for minimally invasive interventions and offers a straightforward administration method. However, non-clinical studies have shown that this approach often results in a significant proportion of transplanted cells being redirected to peripheral organs rather than reaching the intended CNS target [47, 48]. Additionally, the relatively large size of hNSCs (> 8 μm in diameter) poses a risk of pulmonary edema, as these cells are unable to pass through capillaries in a single file like blood cells. Furthermore, while neurodegenerative diseases can compromise BBB integrity, potentially facilitating cell infiltration, this barrier remains a major obstacle to effective IV-based NSC delivery. Hence, we hypothesized that ICV transplantation (directly into the lateral ventricles) could serve as a novel alternative to enhance hNSC biodistribution and neurotrophic factors release throughout the neuraxis by leveraging CSF circulation, which flows from the choroid plexus to the fourth ventricle and into the subarachnoid space [49, 50]. Additionally, it would ideally allow for repeated injections, ensuring continuous delivery of healthy hNSCs, potentially maximizing their therapeutic impact. Of note, ICV transplantation is a technically simple, rapid procedure with a low incidence of complications. In fact, it has already been standardized for intracerebral drug delivery in humans and was recently applied in our clinical study, where up to 24 million cells were safely administered into the ventricles of SPMS patients [17].

Another still open challenge in the context of non-clinical paradigms investigating therapeutic efficacy of stem cells treatments is establishing an effective immunosuppressive regimen. Previous studies from our group [12, 20, 33] extensively demonstrated that a transient low-dose CsA protocol is sufficient to ensure long-term and robust cell engraftment in rats, including SOD1^G93A^ models. However, when applied to SOD1^G93A^ mice, this transient protocol failed to sustain even minimal hNSC survival, with viable transplanted cells detected in only one animal. In this study, we show that only higher doses of hNSCs (1 × 10^6^), combined with prolonged high-dose (30 mg/kg) immunosuppression, enhanced cell survival (with ~ 70% of the animals showing viable grafts), biodistribution, and therapeutic outcomes in ALS mice. Interestingly, cells spread along the ventricles, improving from the low-to-high dose treatment, and reached the central canal of the cervical SC, though none migrated near the thoraco-lumbar tract or into the spinal parenchyma.

While the astro- and micro-gliosis evaluation in the motor cortex was confounded by the gliotic scar-like reaction to the needle, we observed a trend toward higher spinal volume and reduced microgliosis in the cervical region. Under disease conditions, microglia adopt a pro-inflammatory phenotype, further exacerbating neurodegeneration [51]. Although recent studies highlight the dynamic and heterogenous nature of microglial cells, challenging the traditional resting (M2) and activated (M1) phenotypes, it remains clear that microglia undergo pronounced morphological changes from a ramified to an ameboid shape in CNS pathologies [51]. Our study shows that this trend seems to be counteracted following hNSC transplantation, as evidenced by increased proportion of ramified microglial cells in hNSC-treated mice. This evidence was coupled with higher TREM2 levels, a crucial regulator of microglial phagocytic activity with recognized anti-inflammatory properties [52], in the same SC tract. Elevated levels of this protein have been implicated to a not yet fully clarified neuroprotective mechanisms in the inflamed milieu. Both in vitro [53] and in vivo [54] studies have demonstrated decreased TREM2 expression across several neurodegenerative diseases models, including ALS [55]. However, given the limited understanding of TREM2’s function across ALS stages and the increasing evidence highlighting the complexity of microglial phenotypes beyond the conventional M1/M2 paradigm [56], further analyses are required to draw definitive conclusions.

Nonetheless, several preclinical studies describe a delay of clinical symptoms of disease progression (including improved motor outcomes) coupled with reduced microgliosis [12, 57, 58]. Indeed, microgliosis is considered a key histopathological feature of ALS, and possibly a player in its pathogenesis. However, a causal mechanism connecting reduced microgliosis to motor symptoms amelioration still has to be elucidated. In line with current literature, our study describes a transient behavioral improvement, alongside a shift toward a more ramified, resting-like microglial phenotype and a higher TREM2 expression in the cervical spinal cord of hNSC-treated mice. Nevertheless, we acknowledge the complexity of ALS pathology and the limitations of our model, which prevent us from establishing a direct causal relationship. To determine whether microgliosis reduction directly contributes to behavioral improvements in ALS models, more targeted experiments, such as microglia depletion or pharmacological modulation should be performed.

Overall, the observed behavioural and histopathological changes suggest that the local presence of viable cells, rather than direct parenchymal integration, may be sufficient for therapeutic benefits (at least to a certain extent). This aligns with previous studies on mesenchymal stem cell transplantation into the cisterna magna [59, 60] or lumbaris [27] of ALS mice, reinforcing the potential of paracrine effects over direct cell replacement. Further studies should explore whether multiple ICV injections or subsequent injections in different CNS regions (ICV and intrathecal, e.g. cisterna lumbaris) would be more effective compared to a single administration. Finally, the observed limited parenchymal migration may reflect insufficient inflammatory signals required for NSC homing, which may not yet be present at biologically relevant concentrations during early disease stages when we delivered our hNSCs [61]. Nonetheless, our decision to transplant pre-symptomatically was twofold: (i) evidence suggests that upper MNs may undergo pathology prior to clinical symptoms onset, making them early targets in ALS [30–32, 62, 63]; and (ii) early transplantation maximizes the time window for hNSCs within the hosts environment, accounting for their slower inner biological features respect to their murine counterparts and ALS progression in mice. Furthermore, it is known that neurodegeneration and functional decline in ALS patients can precede diagnosis [64]. Thus, early transplantations might extend hNSC therapeutic window and potentially slow down the degeneration of upper MNs. However, investigating the effects of ICV cell delivery at later stages, when inflammation is more robust, may enhance hNSC homing and neuroprotection and be more clinically relevant as ALS diagnosis in humans typically occurs at symptomatic stages.

Inevitably, there are limitations to our study, primarily related to the reactive murine immune response to transplanted cells that severely limit hNSC engraftment. Behavioral and survival analysis, in animals receiving transient CsA treatment, showed no significant differences between transplanted and control groups, aligning with the absence of hNSC survival beyond 15 dpt in this experimental condition. High-dose prolonged immunosuppression and improved hNSC engraftment, overall led to significant behavioral and histopathological outcomes. However, in animals with successful grafts, only ~ 1% of transplanted cells remained in the brain and spinal cord at 40 dpt, potentially limiting therapeutic efficacy. Indeed, the favorable behavioral outcomes were consistent, yet transient, and the reduction of microgliosis was limited to the cervical tract of the spinal cord.

Similar drawbacks have been reported in non-clinical [12, 41] and early-phase clinical trials [14, 65], using intraspinal hNSCs transplant. Although direct comparisons between studies using different animal models and hNSC sources are difficult, the limitations may arise from common factors. These include the time required for hNSCs to engraft and provide protective functions, the rapid disease progression in SOD1^G93A^ models and, as mentioned above, hNSCs underdosing, partly due to rejection [44, 66].

Existing literature suggests that intraparenchymal hNSC transplants provide localized benefits, with no significant effects observed beyond the grafted region [41]. Our approach aimed to overcome this limitation by delivering hNSCs into the CSF circulation, allowing broader biodistribution along the neuraxis. Our findings suggest that while paracrine effects likely play a key role over direct parenchymal integration, their therapeutic impact appears to depend on the local presence of viable cells. In our experiments, huN^+^ cells were identified only in the cervical spinal cord’s central canal, correlating with significant but regionally restricted histological changes. However, given the still limited understanding of hNSC-mediated mechanisms in ALS, we cannot exclude that the observed therapeutic limitations stem from an insufficient number of transplanted cells and excessive dilution of secreted neurotrophic factors rather than the necessity of local cell presence or the need of multiple injections (chronic treatment). Thus, optimizing cell survival and exploring multiple injections of healthy hNSCs remain critical areas for future investigation to fully harness their therapeutic potential.

## Conclusions and future directions

In conclusion, our findings support the potential of hNSC transplantation to mitigate ALS pathology. While intraspinal hNSC transplants have demonstrated benefits in preclinical models and early clinical trials, their broad translation in clinical practice is hindered by the risks associated with extensive laminectomy, especially when delivering high cell doses. Here, we show that ICV transplantation can induce motor function preservation and reduce microgliosis, offering a less invasive alternative to intraparenchymal delivery. Notably, our data align with previous studies [67], suggesting that increased hNSC survival and higher cell doses progressively correlate with enhanced therapeutic effects, reinforcing the clinical relevance of this strategy. Importantly, ICV delivery is a standardized and relatively simple procedure that allows for repeated cell administrations over time, maximizing the therapeutic potential of hNSCs. Furthermore, our recent Phase I study demonstrated the safety of ICV injection of up to 24 million hNSCs in the lateral ventricles of multiple sclerosis patients [17], supporting early-phase clinical investigations of this approach also in ALS. However, given the complexity of the disease, key aspects must be further optimized in preclinical studies to better design truly informative future efficacy trials. These include refining delivery protocols, selecting appropriate animal models, targeting specific CNS regions, assessing sustained improvements across multiple motor tasks, and elucidating the molecular mechanisms underlying hNSC-mediated therapeutic effects. In this regard, our study highlights important challenges, primarily related to the limited survival of hNSCs in SOD1^G93A^ mice and the rapid disease progression, which likely masked their full therapeutic potential. Future studies should explore this approach in more permissive models, such as SOD1^G93A^ rats [12], and investigate putative biomarkers of hNSC activity, including NfL reduction in CSF [68], alongside a more comprehensive evaluation of neuroinflammatory modulation. However, while SOD1^G93A^ rodents are a well-established model for ALS research, SOD1 mutations account for only a small fraction of human ALS cases, and many therapies that showed promise in this model have failed in clinical trials [69]. Hence, as no single animal model fully recapitulate human ALS, integrating non-clinical data with early-phase clinical trials is paramount to fully harness the therapeutic potential of hNSCs. In this context, an ongoing clinical trial for ALS patients (NCT06344260) aims to establish the maximum tolerated dose of hNSCs delivered via ICV while exploring initial efficacy. Future research should leverage advanced in vitro [70] and in vivo [71] models, incorporating both familial and sporadic ALS cases, and explore combinatorial therapeutic approaches to unravel the molecular underpinnings of hNSC-mediated neuroprotection and repair.

## Electronic supplementary material

Below is the link to the electronic supplementary material.

Supplementary Material 1: Histological analysis of the cervical spinal cord in SOD1^G93A^ mice following hNSC ICV transplantation: additional microglial markers. a) Representative images of microglial markers within the ventral horn of the cervical spinal cord of hNSC- vs. HBSS-treated SOD1^G93A^ mice. A magnification of a transient cell expressing all the markers in shown on the right| IBA1, green; CD206, white; CD86, red; DAPI, blue. b) Semi-quantitative analysis the fluorescence intensity (MGV) of the microglial markers IBA1, CD86, and CD206 hNSC- vs. HBSS-treated SOD1^G93A^ mice (*n* = 3/group). c) Count (%) of the IBA1^+^ cells expressing CD86, CD206 or CD86/CD206 in hNSC- vs. HBSS-treated SOD1^G93A^ mice.

## Data Availability

All data generated or analysed during this study are included in this published article. Data analysed will be made available from the corresponding authors on reasonable request.

## Declarations

### Generative AI and AI-assisted technologies in the writing process

During the preparation of this work the authors used ChatGPT to check grammar. After using this tool/service, the authors reviewed and edited the content as needed and take full responsibility for the content of the publication.
