# Supplementary figures and images for: Safety and efficacy evaluation of intracerebroventricular human neural stem cell transplantation in SOD1 mice as a novel approach for ALS
Source: J Transl Med. 2025 May 9;23:529. doi: 10.1186/s12967-025-06529-9 (PMC12065241; doi:10.1186/s12967-025-06529-9)

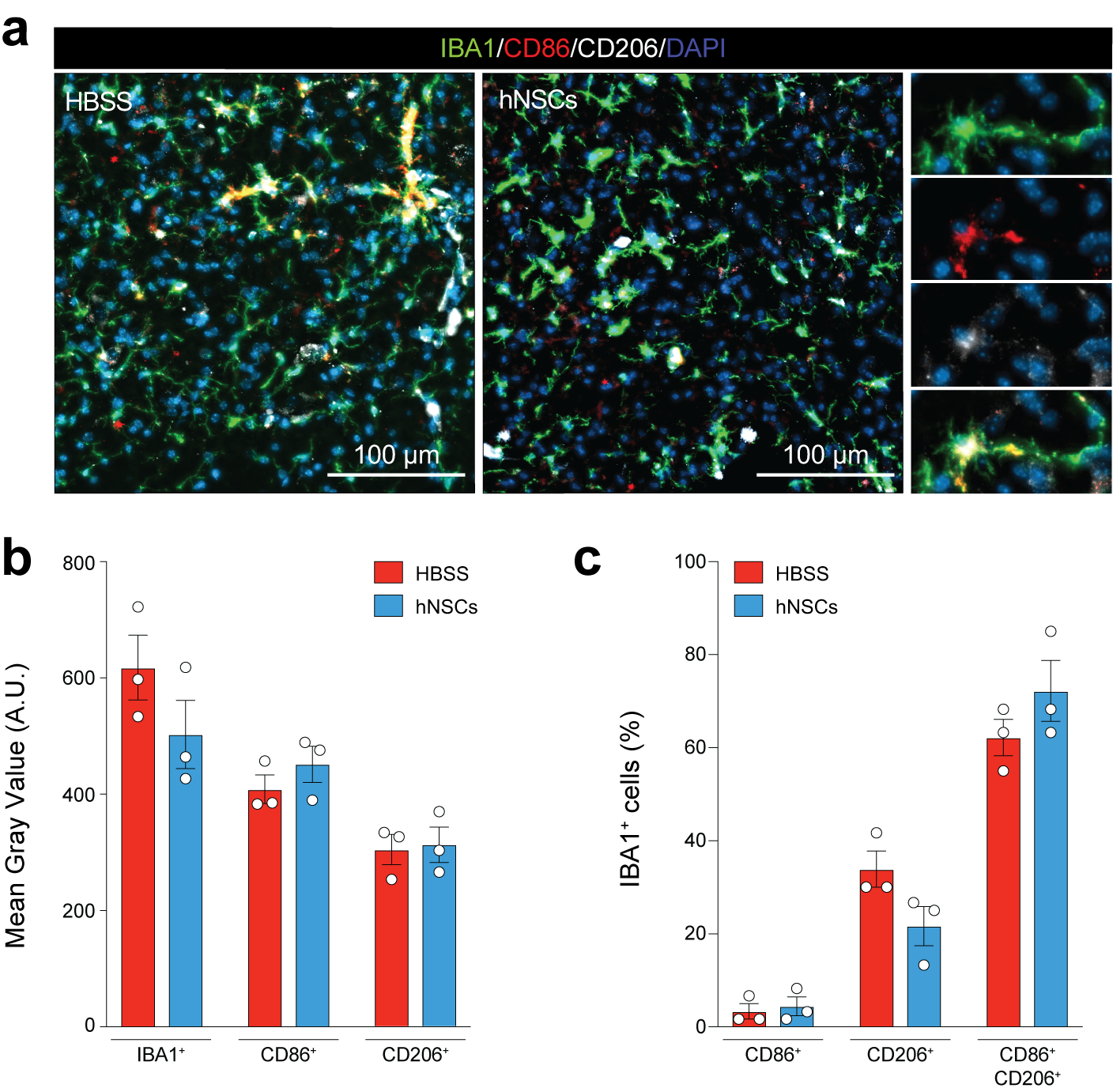

Supplement: Supplementary file 1 — Supplementary Material 1: Histological analysis of the cervical spinal cord in SOD1G93A mice following hNSC ICV transplantation: additional microglial markers. a) Representative images of microglial markers within the ventral horn of the cervical spinal cord of hNSC- vs. HBSS-treated SOD1G93A mice. A magnification of a transient cell expressing all the markers in shown on the right| IBA1, green; CD206, white; CD86, red; DAPI, blue. b) Semi-quantitative analysis the fluorescence intensity (MGV) of the microglial markers IBA1, CD86, and CD206 hNSC- vs. HBSS-treated SOD1G93A mice (n = 3/group). c) Count (%) of the IBA1+ cells expressing CD86, CD206 or CD86/CD206 in hNSC- vs. HBSS-treated SOD1G93A mice. [file 12967_2025_6529_MOESM1_ESM.tif]
